# Comparison of Multivariable Logistic Regression and Machine Learning Models for Predicting Bronchopulmonary Dysplasia or Death in Very Preterm Infants

**DOI:** 10.3389/fped.2021.759776

**Published:** 2021-12-07

**Authors:** Faiza Khurshid, Helen Coo, Amal Khalil, Jonathan Messiha, Joseph Y. Ting, Jonathan Wong, Prakesh S. Shah

**Affiliations:** ^1^Department of Pediatrics, Queen's University, Kingston, ON, Canada; ^2^Centre for Advanced Computing, Queen's University, Kingston, ON, Canada; ^3^Smith School of Business, Queen's University, Kingston, ON, Canada; ^4^Department of Pediatrics, University of British Columbia, Vancouver, BC, Canada; ^5^Department of Paediatrics, University of Toronto, Toronto, ON, Canada; ^6^Institute of Health Policy, Management and Evaluation, University of Toronto, Toronto, ON, Canada; ^7^Lunenfeld-Tanenbaum Research Institute, Sinai Health, Toronto, ON, Canada

**Keywords:** bronchopulmonary dysplasia, chronic lung disease, prediction, machine learning, discrimination, calibration

## Abstract

Bronchopulmonary dysplasia (BPD) is the most prevalent and clinically significant complication of prematurity. Accurate identification of at-risk infants would enable ongoing intervention to improve outcomes. Although postnatal exposures are known to affect an infant's likelihood of developing BPD, most existing BPD prediction models do not allow risk to be evaluated at different time points, and/or are not suitable for use in ethno-diverse populations. A comprehensive approach to developing clinical prediction models avoids assumptions as to which method will yield the optimal results by testing multiple algorithms/models. We compared the performance of machine learning and logistic regression models in predicting BPD/death. Our main cohort included infants <33 weeks' gestational age (GA) admitted to a Canadian Neonatal Network site from 2016 to 2018 (*n* = 9,006) with all analyses repeated for the <29 weeks' GA subcohort (*n* = 4,246). Models were developed to predict, on days 1, 7, and 14 of admission to neonatal intensive care, the composite outcome of BPD/death prior to discharge. Ten-fold cross-validation and a 20% hold-out sample were used to measure area under the curve (AUC). Calibration intercepts and slopes were estimated by regressing the outcome on the log-odds of the predicted probabilities. The model AUCs ranged from 0.811 to 0.886. Model discrimination was lower in the <29 weeks' GA subcohort (AUCs 0.699–0.790). Several machine learning models had a suboptimal calibration intercept and/or slope (k-nearest neighbor, random forest, artificial neural network, stacking neural network ensemble). The top-performing algorithms will be used to develop multinomial models and an online risk estimator for predicting BPD severity and death that does not require information on ethnicity.

## Introduction

Bronchopulmonary dysplasia (BPD), a form of chronic lung disease, is the most common morbidity among very preterm neonates (i.e., those born before 33 weeks of gestation) (1). Studies have demonstrated long-term respiratory (2–4), neurodevelopmental (5–7), and cardiovascular sequelae among survivors (8, 9). Although the pathways leading to BPD are not fully understood (10), both prenatal and postnatal exposures are associated with its development (1).

BPD is commonly defined as the need for oxygen and/or respiratory support at 36 weeks' postmenstrual age (11). The interval between very preterm delivery and the establishment of a diagnosis provides an opportunity for initiating disease modification interventions. Clinical prediction models can support the identification of at-risk patients in order to appropriately target such interventions. The Eunice Kennedy Shriver National Institute of Child Health and Human Development (NICHD) hosts an online BPD risk estimator that allows users to predict the probability of mild, moderate and severe BPD, and death, on postnatal days 1, 3, 7, 14, 21, and 28 (12). This risk estimator has not been validated in the Canadian population and was developed using data from 2000 to 2004 (13). The clinical management of very preterm infants, including ventilation support, has evolved markedly since that time (14, 15). Moreover, the estimator's functionality is limited in ethno-diverse populations: ethnicity is a required input and the only response options are White, Black or Hispanic. Most other BPD prediction models only allow an infant's risk to be estimated at one point in time [reviewed in (16, 17)] and therefore have limited clinical utility, given that postnatal exposures are known to affect the likelihood of developing BPD (1).

While regression-based methods have traditionally dominated the field of predictive modeling—with existing BPD prediction models being no exception (16)—machine learning algorithms are also available for this purpose. Leo Breiman, in a seminal article titled “Statistical Modeling: The Two Cultures,” outlines a rationale for adopting a more diverse set of tools (specifically, machine learning) when using data in research applications, including predictive modeling (18). Boulesteix and Schmid further note, “It is not easy to foresee which method will perform better on a particular dataset (…) In this perspective, there is no reason to restrict to a single prediction method if the goal is to achieve good prediction accuracy” [(19), p. 589]. It is prudent, therefore, to adopt a more comprehensive approach when developing clinical prediction models, one in which multiple algorithms/models are compared to identify the top-performing one(s).

We are aware of only one published report that compared a range of machine learning algorithms with regression-based methods for predicting BPD (20). The investigators focused exclusively on discrimination performance (i.e., how well a model differentiates between those who do and do not experience the outcome) and did not examine how well their models' predictions matched the observed outcome (known as calibration) (21). Both measures are critical when assessing performance, as the predicted risks may be unreliable if a model exhibits good discrimination but poor calibration (22). As such, their report provides an incomplete comparison of machine learning and regression-based methods for predicting BPD.

Our objectives were to compare the performance of machine learning and logistic regression models in predicting the composite outcome of BPD/death on days 1, 7, and 14 of an infant's stay in the neonatal intensive care unit (NICU) and to identify the top-performing algorithms/models. These will be used in future work to develop an online tool for predicting BPD severity and death that does not require information on the infant's ethnicity, using data from a Canadian cohort exposed to contemporary respiratory support practices.

## Materials and Methods

The study protocol was reviewed for ethical compliance by the Queen's University Health Sciences and Affiliated Teaching Hospitals Research Ethics Board and the Mount Sinai Hospital Research Ethics Board. We followed the Transparent Reporting of a multivariable prediction model for Individual Prognosis Or Diagnosis (TRIPOD) recommendations (23).

### Data Source and Cohort

We used a database maintained by the Canadian Neonatal Network (CNN) that captures >90% of admissions to tertiary NICUs across Canada (*n* = 31 at the time of this study) and includes information on maternal characteristics, infant demographics, delivery room interventions, daily interventions, and infant outcomes (24). Trained personnel abstract data from patient charts into an electronic database with built-in error checking. An internal audit revealed high levels of agreement (>95%) between the original and the re-abstracted data (25).

We included infants born before 33 weeks of gestation who were admitted between January 1, 2016 and December 31, 2018. Infants were excluded if they were transferred to a lower level of care or discharged home within 24 h of admission; were moribund on admission or died on their first day in the NICU; had a severe congenital anomaly; were admitted on postnatal day 3 or later (where the day of delivery was considered postnatal day 1); were discharged within 14 days of admission for reasons other than death, or if the discharge date was missing; or if their outcome status could not otherwise be determined. Infants who died on or before days 7 and 14 of their NICU stay were further excluded from the Day 7 and Day 14 models, respectively. We did not perform sample size calculations.

### Outcome and Predictors

We examined the composite outcome of BPD/death prior to discharge from the tertiary care unit. BPD was defined as receipt of any of the following supports at 36 weeks' postmenstrual age or at discharge from the NICU, whichever occurred earlier: >21% oxygen, high-frequency ventilation, intermittent positive pressure ventilation, non-invasive ventilation, continuous positive airway pressure, or high flow air/oxygen at flow rate >1.5 liters/minute. All other eligible infants were classified as “Survived to NICU discharge without BPD.” An initial list of predictors was created by reviewing the literature on risk factors for BPD (26–30) and existing BPD prediction models [reviewed in (16, 17)]. The final predictors were chosen based on availability in the CNN database and are listed in Table 1.

**Table 1:** Variables entered in models to predict bronchopulmonary dysplasia or death prior to NICU discharge among very preterm infants on Days 1, 7, and 14 of admission to Canadian NICUs, 2016–2018.

**Predictor**	**Possible values^**a**^**	**Day 1 models**	**Day 7 models**	**Day 14 models**
Inborn	Yes (born in hospital where NICU located) No (transferred in) Missing	Y	Y	Y
Sex	Boy Girl Missing	Y	Y	Y
Gestational age (weeks and days)	<33.0 weeks	Y	Y	Y
Small for gestational age (birthweight <10th percentile for gestational age and sex)	Yes No Missing	Y	Y	Y
SNAPPE-II score (31) (Score for Neonatal Acute Physiology with Perinatal Extension-II; newborn illness severity score based on 9 vital signs and laboratory test results measured in first 12 h of admission to NICU, where higher scores indicate higher illness severity)	0–162 Missing	Y	Y	Y
Hypertension	Pre-existing Gestational Yes, timing unknown No Missing	Y	Y	Y
Complete course of antenatal steroids in week preceding delivery	Yes No (partial course, or none) Missing	Y	Y	Y
Preterm premature rupture of membranes	Yes (≥24 h between rupture of membranes and birth) No (<24 h) Missing	Y	Y	Y
Mode of delivery	Caesarean Vaginal Missing	Y	Y	Y
Delivery room resuscitation requiring intubation	Yes (intubation, chest compression, and/or epinephrine administered in delivery room) No Missing	Y	Y	Y
Surfactant administered on or before day of prediction (e.g., for Day 1 models, “Yes” if surfactant administered on day admitted to NICU; for Day 7 models, “Yes” if administered on or before day 7 of NICU stay)	Yes No	Y	Y	Y
Nitric oxide administered on first day of NICU stay	Yes No	Y	N	N
Number of days on nitric oxide up to and including day of prediction	0–7 (Day 7 models) 0–14 (Day 14 models)	N	Y	Y
Inotropes administered on first day of NICU stay	Yes No	Y	N	N
Number of days on inotropes up to and including day of prediction	0–7 (Day 7 models) 0–14 (Day 14 models)	N	Y	Y
HFV or IPPV on first day of NICU stay	Yes No	Y	N	N
Number of days of HFV or IPPV up to and including day of prediction	0–7 (Day 7 models) 0–14 (Day 14 models)	N	Y	Y
NIV or CPAP on first day of NICU stay	Yes No	Y	N	N
Number of days of NIV or CPAP up to and including day of prediction	0–7 (Day 7 models) 0–14 (Day 14 models)	N	Y	Y
Culture-confirmed sepsis on or before Day 7 of NICU stay	Yes No	N	Y	N
Culture-confirmed sepsis on or before Day 14 of NICU stay	Yes No	N	N	Y

*CPAP, Continuous positive airway pressure*.

*HFV, High-frequency ventilation*.

*IPPV, Intermittent positive pressure ventilation*.

*NICU, Neonatal intensive care unit*.

*NIV, Non-invasive ventilation*.

a *Prior to one-hot encoding, categorizing continuous variables where violations of assumption of linearity with respect to logit of outcome detected, and imputing missing values*.

### Data Preprocessing

The study dataset was imported into Python version 3.6 (Python Software Foundation, https://www.python.org/).

#### Managing Continuous Variables

In logistic regression, the continuous predictors are assumed to be linearly related to the logit of the outcome (32). Preliminary analyses revealed that two continuous predictors—gestational age (GA) and the Score for Neonatal Acute Physiology with Perinatal Extension-II (SNAPPE-II) score [a newborn illness severity score (31)]—were two of the strongest predictors of BPD/death. We verified whether the assumption of linearity was violated for these variables by first entering all the predictors into a logistic regression model. The same set of predictors, as well as a product term for GA and its natural logarithm, were entered into a second model. Because the product term was statistically significant (32), separate categories for each GA in completed weeks were created. We repeated this process for the SNAPPE-II score. The product term was not significant, and so SNAPPE-II score was modeled as a continuous variable. For simplicity, and because the current aim was not to develop clinical prediction models *per se* but rather to compare the performance of models trained using different approaches, linearity was assumed for all other non-categorical variables. The continuous predictors were then standardized so that their values ranged between 0 and 1. Each predictor was thus given equal consideration by the k-nearest neighbor algorithm during the model training phase; this algorithm calculates the distance between data points to make its predictions and thus is sensitive to variable scaling (33).

#### One-Hot Encoding

All non-binary categorical predictors (e.g., hypertension; see Table 1) were transformed into multiple binary variables through one-hot encoding [a process similar to creating dummy variables in regular statistical modeling, except that in the case of *n* categories one-hot encoding will create *n* variables, each of whose values are coded as “0” or “1” (34)].

### Algorithms/Ensembles and Prediction Time Points

There is no consensus as to whether logistic regression should be considered a machine learning algorithm (35). For our purposes, we refer to unpenalized logistic regression as “standard logistic regression” and consider penalized logistic regression to be a machine learning algorithm. In penalized regression, a penalty term is added to the model to reduce overfitting (36).

In addition to penalized logistic regression, we examined three other commonly used machine learning algorithms [support vector machine (37), k-nearest neighbor (38), artificial neural network (39)] and three ensemble methods [random forest (40), soft voting ensemble, stacking neural network ensemble]. A description of the models is provided in Supplementary Table 1.

### Model Training and Internal Validation

We used two methods to train and internally validate the models: 10-fold cross-validation and a training-test split (41). For each set of models (Days 1, 7, 14), stratified random sampling was used to select 80% of records within each outcome class (BPD/death, survived to NICU discharge without BPD) to form the training/cross-validation dataset. The remaining 20% of records formed the test dataset.

With each method, missing values were imputed separately for the training and test/validation data (i.e., in the training-test split procedure, missing values were imputed separately for a) the 80% of records forming the training dataset and b) the 20% of records forming the test dataset; and in the 10-fold cross-validation procedure, the missing values in the training dataset were imputed at the beginning of each cycle separately for a) the nine folds comprising the training data and b) the tenth fold comprising the validation data) using the IterativeImputer procedure from Python's Sklearn library (42). This procedure sequentially treats each variable with missing values as the dependent variable and regresses it on all the other variables to impute values for the missing data. “Iterative” refers to the fact that the procedure is repeated multiple times (in our case, we used the default value of 10), under the assumption that this will result in increasingly more accurate and stable estimates of the missing values (43). Only the values imputed in the final iteration are used to fit the models, and therefore IterativeImputer is considered a single imputation procedure (42).

Discrimination performance was assessed by the area under the curve (AUC) of the receiver operating characteristic curve (44) for the models developed using the 10-fold cross-validation and the training-test split procedures. Model calibration was evaluated in the test dataset using methods that are described further on in the section outlining the training-test split procedure.

#### 10-Fold Cross-Validation

Stratified random sampling was again used to divide the training/cross-validation dataset (i.e., the dataset containing 80% of the total records) into ten folds. K-fold cross-validation (in our case, k = 10) is a procedure whereby multiple models are generated and validated in multiple subsets of the data, with each record used k-1 times for training and exactly once for validation (45). In each cycle of our procedure (where 10 cycles = 1 iteration), the models were trained on nine of the folds. All the predictors were included, i.e., no variable selection techniques were used. Interactions were not explicitly tested. {Note, however, that certain algorithms automatically model interactions between predictors. These include tree-based methods [e.g., random forests (46)] and artificial neural networks (47).} Each cycle generated an AUC when the model was validated in the holdout fold. One iteration of the procedure thus generated 10 AUCs.

Machine learning algorithms use hyperparameters to search for the optimal solution during model training (48). An example of a hyperparameter is the “k” in k-nearest neighbor, which specifies the number of data points that are used in the training dataset to classify new observations (38). Unlike the parameters of a logistic regression model, hyperparameters are not estimated. Instead, software packages offer default values. The analyst can also manually set the values or use a tuning procedure to configure them (49). The optimal hyperparameter values for our models (see Supplementary Table 1) were determined in the initial iterations of the 10-fold cross-validation procedure. The default settings were used during the first iteration. In subsequent iterations, the parameters were “tweaked” to examine the effect on the AUC. This process was repeated until the hyperparameters were tuned to yield the highest AUCs. No hyperparameters were tuned for the standard logistic regression models, with the “penalty” hyperparameter set to “none.”

Once the hyperparameters were tuned, a final iteration of the 10-fold cross-validation procedure was performed. The AUCs from those final ten cycles were averaged.

#### Training-Test Split

The algorithms were then run on the full training/cross-validation dataset using the optimized hyperparameter settings. The discrimination performance of the resulting models was evaluated in the test dataset and we generated 95% confidence intervals for those AUCs using the formula provided by Hanley and McNeil (44). We also calculated sensitivity, specificity, as well as positive and negative predictive values and 95% confidence intervals by entering the number of true positives and negatives and false positive and negatives in the test dataset—using the default threshold of probability of BPD/death ≥0.5 to denote “test positive”—into an online calculator (https://www.medcalc.org/calc/diagnostic_test.php). The predicted probabilities of BPD/death for records in the test dataset were saved. Model calibration was examined by comparing the mean predicted probabilities to the observed proportion of BPD/death. Calibration intercepts and slopes were also estimated (50, 51). The calibration intercept has a target value of 0 and indicates whether a model systematically overestimates (negative intercept) or underestimates (positive intercept) the risk (22, 52, 53). It was obtained by fitting a univariate logistic regression model with BPD/death as the dependent variable and the log-odds of the predicted probability as an offset variable (51). The calibration slope has a target value of 1. A slope <1 indicates that the predicted probabilities are too extreme (i.e., too high for those at high risk and too low for those at low risk), whereas a slope >1 indicates a model's predictions are too high for those at low risk and too low for those at high risk (22, 53). The calibration slope was estimated by fitting a second logistic regression model with BPD/death as the dependent variable and the log odds of the predicted probability as the independent variable, with the intercept set to the value estimated in the previous step (50, 51, 54). The predicted probabilities and observed outcomes were then imported into SAS Enterprise Guide v. 7.13 (SAS Institute Inc., Cary, NC). We used the SGPLOT procedure to fit loess curves for the observed and predicted probabilities.

Machine learning includes methods for assigning scores to indicate the relative importance of each variable in predicting the outcome of interest. We used random forests to generate these so-called feature importance scores (55), which were decimal figures that summed to 1.

### Sensitivity/Subgroup Analyses

To examine whether the decision to categorize GA had any impact on the discrimination performance of the machine learning models, GA was retained in continuous form and the 10-fold cross-validation procedure was repeated.

A 2019 commentary observed that “the critical user of prediction models should look for evidence that the reported overall accuracy of models still applies for subgroups of patients with very different input or outcome prevalence.” (56). The incidence of BPD is much higher in younger GA groups, particularly among those born prior to 29 weeks of gestation (57). Accordingly, we also trained and validated models using data from this subgroup of infants.

## Results

**Figure 1:**
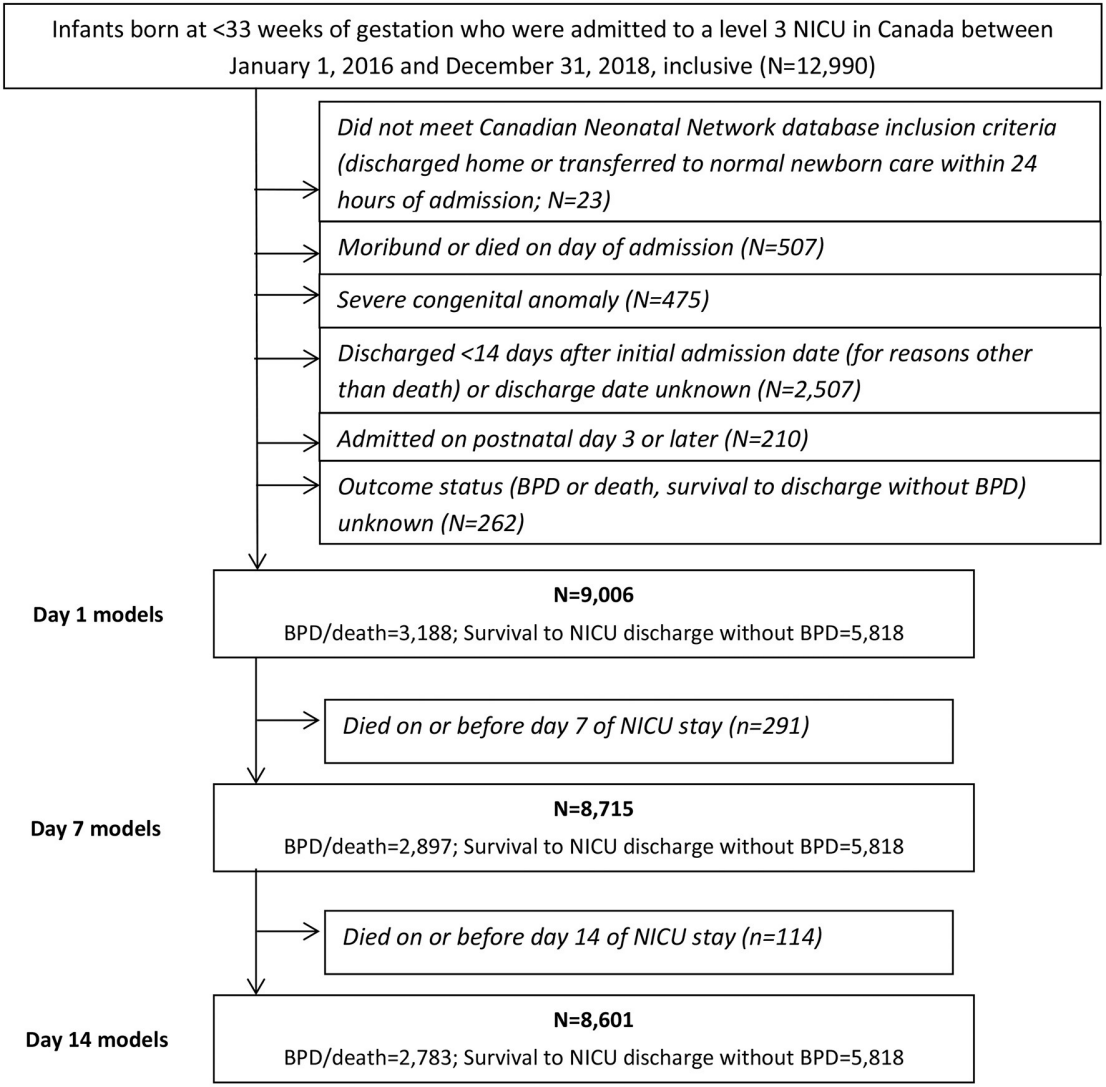
Selection of study cohort.

A total of 12,990 neonates born prior to 33 weeks of gestation were admitted to a tertiary-care NICU in Canada between January 1, 2016 and December 31, 2018. After the exclusion criteria were applied, the main study cohort numbered 9,006, approximately one-third [*n* = 3,188 (35%)] of whom developed BPD or died during their NICU stay (see Figure 1). These infants formed the cohort for the Day 1 models. Their characteristics are shown in Table 2. After excluding deaths on or prior to days 7 and 14 of admission, the cohorts for the Day 7 and Day 14 models numbered 8,715 (*n* = 2,897 (33%) BPD/death) and 8,601 (*n* = 2,783 (32%) BPD/death), respectively. The corresponding numbers for the <29 weeks' GA subcohort were 4,246 (*n* = 2,510 (59%) BPD/death); 4,000 (*n* = 2,264 (57%) BPD/death); and 3,899 (*n* = 2,163 (55%) BPD/death).

**Table 2:** Characteristics of infants born at <33 weeks of gestation who were admitted to a Canadian tertiary-care NICU from 2016 to 2018 and whose data were included in models to predict bronchopulmonary dysplasia (BPD) or death prior to NICU discharge.

	**Missing values**	**BPD or death** **(*n* = 3,188)**	**Survived to NICU discharge without BPD** **(*n* = 5,818)**

	***n*** **(%**^**a**^**)**	***n*** **(Valid %**^**b**^**) or** **Mean (SD)** **Median (range; IQR)**	***n*** **(Valid %**^**b**^**) or** **Mean (SD)** **Median (range; IQR)**
Inborn	<5 (<0.05)	2,757 (86)	5,180 (89)
Male	8 (0.09)	1,785 (56)	3,143 (54)
Gestational age, completed weeks	0	26.5 (2.4) 26 (21–32; 25–28)	29.5 (2.1) 30 (23–32; 28–31)
Small for gestational age	9 (0.10)	441 (14)	487 (8.4)
SNAPPE-II score (31)	115 (1.3)	16.6 (14.1) 14 (0–84; 7–24)	6.1 (8.3) 0 (0–59; 0–9)
Hypertension	217 (2.4)		
Pre-existing		116 (3.7)	198 (3.5)
Gestational		430 (14)	935 (16)
Yes, timing unknown		10 (0.32)	16 (0.28)
Complete case of antenatal steroids in week preceding delivery	97 (1.1)	1,233 (39)	2,219 (39)
Preterm premature rupture of membranes	399 (4.4)	768 (25)	1,271 (23)
Caesarean delivery	18 (0.20)	2,007 (63)	3,639 (62)
Delivery room resuscitation requiring intubation	79 (0.88)	1,556 (49)	853 (15)
Surfactant	0		
First day of NICU stay		1,961 (62)	1,485 (26)
On or before day 7 of NICU stay		2,384 (75)	1,940 (33)
On or before day 14 of NICU stay		2,393 (75)	1,943 (33)
Nitric oxide	0		
First day of NICU stay		192 (6.0)	43 (0.74)
Frequency up to and including day 7 of NICU stay, days		0.33 (1.1) 0^c^ (0–7)	0.07 (0.50) 0^c^ (0–7)
Frequency up to and including day 14 of NICU stay, days		0.51 (1.7) 0^c^ (0–14)	0.07 (0.53) 0^c^ (0–9)
Inotropes	0		
First day of NICU stay		325 (10)	108 (1.9)
Frequency up to and including day 7 of NICU stay, days		0.6 (1.4) 0^c^ (0–7)	0.18 (0.73) 0^c^ (0–7)
Frequency up to and including day 14 of NICU stay, days		0.9 (2.1) 0^c^ (0–14)	0.22 (0.94) 0^c^ (0–9)
High-frequency ventilation or intermittent positive pressure ventilation	0		
First day of NICU stay		2,060 (65)	1,441 (25)
Frequency up to and including day 7 of NICU stay, days		4.4 (2.8) 6 (0–7; 2–7)	2.0 (2.4) 1 (0–7; 0–3)
Frequency up to and including day 14 of NICU stay, days		8.0 (5.6) 9 (0–14; 2–14)	2.9 (4.1) 1 (0–14; 0–4)
Non-invasive ventilation or continuous positive airway pressure	0		
First day of NICU stay		984 (31)	3,496 (60)
Frequency up to and including day 7 of NICU stay, days		2.5 (2.8) 1 (0–7; 0–5)	4.6 (2.5) 5.0 (0–7; 3–7)
Frequency up to and including day 14 of NICU stay, days		5.8 (5.5) 5 (0–14; 0–12)	9.3 (4.6) 11 (0–14; 6–14)
Culture-confirmed sepsis	0		
On or before day 7 of NICU stay		228 (7.2)	132 (2.3)
On or before day 14 of NICU stay		483 (15)	255 (4.4)

*The data in this table describe the cohort used to develop the Day 1 prediction models*.

*IQR, Interquartile range*.

*NICU, Neonatal intensive care unit*.

*SD, Standard deviation*.

a *Calculated using denominator of n = 9,006*.

b *Denominator excludes missing values*.

c *IQR could not be calculated*.

Table 3 provides the AUCs from the 10-fold cross-validation and the training-test split procedures. In the main cohort, the AUCs ranged from 0.811 to 0.862 for the Day 1 models, 0.812 to 0.886 for the Day 7 models, and 0.815 to 0.884 for the Day 14 models. Discrimination performance was lower in the <29 weeks' GA subcohort: 0.699–0.782 for the Day 1 models, 0.706–0.783 for the Day 7 models, and 0.708–0.790 for the Day 14 models. The average AUCs from the 10-fold cross-validation procedure and the AUCs obtained when the models were run on the test dataset were generally similar (e.g., 0.861 and 0.860 for the Day 1 standard logistic regression models, <33 weeks' GA cohort).

**Table 3:** Area under the curve (AUC) for models predicting bronchopulmonary dysplasia or death prior to NICU discharge at three time points (Days 1, 7, and 14 of NICU stay) among infants born at <33 weeks and <29 weeks of gestation who were admitted to Canadian tertiary-care NICUs, 2016–2018.

	**<33 weeks**						**<29 weeks**					
	**Day 1**		**Day 7**		**Day 14**		**Day 1**		**Day 7**		**Day 14**	
**Model**	**10-fold cross-validation**	**Test dataset** **(95% CI)**	**10-fold cross-validation**	**Test dataset** **(95% CI)**	**10-fold cross-validation**	**Test dataset** **(95% CI)**	**10-fold cross-validation**	**Test dataset** **(95% CI)**	**10-fold cross-validation**	**Test dataset** **(95% CI)**	**10-fold cross-validation**	**Test dataset** **(95% CI)**
Standard Logistic Regression (LR)	0.861	0.860 (0.840–0.880)	0.884	0.884 (0.865–0.903)	0.877	0.878 (0.858–0.898)	0.779	0.782 (0.752–0.812)	0.776	0.783 (0.752–0.814)	0.776	0.790 (0.759–0.821)
Penalized LR	0.861	0.861 (0.841–0.881)	0.884	0.884 (0.865–0.903)	0.878	0.879 (0.859–0.899)	0.781	0.780 (0.750–0.810)	0.777	0.782 (0.751–0.813)	0.775	0.790 (0.759–0.821)
Support Vector Machine	0.830	0.837 (0.816–0.858)	0.859	0.861 (0.841–0.881)	0.853	0.858 (0.837–0.879)	0.758	0.750 (0.718–0.782)	0.737	0.756 (0.723–0.789)	0.768	0.772 (0.740–0.804)
K-Nearest Neighbor	0.814	0.811 (0.789–0.833)	0.812	0.822 (0.799–0.845)	0.815	0.817 (0.794–0.840)	0.724	0.719 (0.685–0.753)	0.707	0.706 (0.671–0.741)	0.708	0.716 (0.681–0.751)
Artificial Neural Network	0.862	0.859 (0.839–0.879)	0.871	0.881 (0.862–0.900)	0.877	0.872 (0.852–0.892)	0.772	0.780 (0.750–0.810)	0.758	0.774 (0.742–0.806)	0.752	0.769 (0.737–0.801)
Random Forest	0.817	0.819 (0.797–0.841)	0.851	0.854 (0.833–0.875)	0.863	0.857 (0.836–0.878)	0.721	0.699 (0.664–0.734)	0.737	0.725 (0.691–0.759)	0.753	0.760 (0.727–0.793)
Soft Voting Ensemble	0.861	0.860 (0.840–0.880)	0.880	0.882 (0.863–0.901)	0.881	0.879 (0.859–0.899)	0.777	0.774 (0.743–0.805)	0.772	0.775 (0.743–0.807)	0.775	0.787 (0.756–0.818)
Stacking Neural Network Ensemble	0.862	0.862 (0.843–0.881)	0.886	0.885 (0.866–0.904)	0.884	0.878 (0.858–0.898)	0.758	0.775 (0.744–0.806)	0.770	0.772 (0.740–0.804)	0.766	0.772 (0.740–0.804)

*NICU, Neonatal intensive care unit*.

For infants born prior to 33 weeks of gestation, the AUCs when GA was modeled as a categorical (Table 3) and continuous variable (Supplementary Table 2) were also generally similar. The differences were more pronounced in the <29 weeks' GA subcohort, with the biggest difference in favor of categorizing GA observed for the Day 1 k-nearest neighbor model (average AUC = 0.692 when modeled as a continuous variable vs. average AUC = 0.724 when categorized) and the biggest difference in favor of modeling it as a continuous variable observed in the Day 7 support vector machine model (average AUC = 0.777 when modeled as a continuous variable vs. average AUC = 0.737 when categorized).

Tables 4, 5 provide the model calibration statistics. The calibration slopes for the k-nearest neighbor and random forest models were significantly lower than the target value of 1, indicating that their predictions were too extreme (note that the models to determine the calibration intercepts and slopes for the Day 7 and Day 14 k-nearest neighbor models did not converge for the main cohort, and so those data are not provided). In contrast, the calibration slopes for the artificial neural network and stacking neural network ensemble models were often significantly higher than 1, meaning that their predictions tended to be too high for those at low risk and too low for those at high risk.

**Table 4:** Calibration of models predicting bronchopulmonary dysplasia or death prior to NICU discharge at three time points (Days 1, 7, and 14 of NICU stay) among infants born at **<33 weeks of gestation** who were admitted to Canadian tertiary-care NICUs in 2016–2018, as assessed in test dataset.

	**Day 1** ***n*** **=** **1,802**				**Day 7** ***n*** **=** **1,743**				**Day 14** ***n*** **=** **1,721**			
	**Observed proportion BPD/death** **(*n =* 638)**	**Mean predicted probability BPD/death**	**Calibration intercept** **(95% CI)**	**Calibration slope** **(95% CI)**	**Observed proportion BPD/death** **(*n =* 579)**	**Mean predicted probability BPD/death**	**Calibration intercept** **(95% CI)**	**Calibration slope** **(95% CI)**	**Observed proportion BPD/death** **(*n =* 557)**	**Mean predicted probability BPD/death**	**Calibration intercept (95% CI)**	**Calibration slope** **(95% CI)**
Standard Logistic Regression (LR)	0.354	0.343	0.08 (−0.04, 0.20)	1.05 (0.96, 1.13)	0.332	0.331	0.006 (−0.12, 0.13)	1.08 (0.98, 1.18)	0.324	0.315	0.07 (−0.06, 0.20)	1.04 (0.94, 1.14)
Penalized LR	0.354	0.343	0.08 (−0.04, 0.20)	1.07 (0.98, 1.16)	0.332	0.331	0.007 (−0.12, 0.13)	1.10 (1.00, 1.20)	0.324	0.315	0.07 (−0.06, 0.20)	1.06 (0.97, 1.16)
Support Vector Machine	0.354	0.347	0.04 (−0.07, 0.16)	1.07 (0.98, 1.16)	0.332	0.331	0.008 (−0.11, 0.13)	1.10 (1.01, 1.19)	0.324	0.316	0.05 (−0.07, 0.18)	1.04 (0.96, 1.13)
K-Nearest Neighbor	0.354	0.337	7.61 (5.71, 9.51)	0.36 (0.35–0.37)	0.332	0.329	—^a^	—^b^	0.324	0.309	—^a^	—^b^
Artificial Neural Network	0.354	0.346	0.05 (−0.07, 0.16)	1.16 (1.06, 1.27)	0.332	0.338	−0.04 (−0.16, 0.09)	1.25 (1.13, 1.37)	0.324	0.319	0.04 (−0.09, 0.16)	1.08 (0.98, 1.18)
Random Forest	0.354	0.348	0.05 (−0.08, 0.18)	0.35 (0.30, 0.40)	0.332	0.333	−0.006 (−0.14, 0.13)	0.46 (0.40, 0.52)	0.324	0.319	0.04 (−0.09, 0.18)	0.60 (0.53, 0.67)
Soft Voting Ensemble	0.354	0.346	0.05 (−0.07, 0.17)	1.10 (1.01, 1.20)	0.332	0.327	0.04 (−0.09, 0.16)	1.15 (1.05–1.25)	0.324	0.319	0.04 (−0.09, 0.16)	1.11 (1.01, 1.21)
Stacking Neural Network Ensemble	0.354	0.365	−0.07 (−0.19, 0.04)	1.10 (1.00, 1.19)	0.332	0.333	−0.003 (−0.13, 0.12)	1.16 (1.06–1.26)	0.324	0.327	−0.02 (−0.15, 0.10)	1.17 (1.07, 1.28)

*NICU, Neonatal intensive care unit*.

a *Model did not converge*.

b *Calibration slope could not be estimated (see preceding footnote)*.

**Table 5:** Calibration of models predicting bronchopulmonary dysplasia or death prior to NICU discharge at three time points (Days 1, 7, and 14 of NICU stay) among infants born at **<29 weeks of gestation** who were admitted to Canadian tertiary-care NICUs in 2016–2018, as assessed in test dataset.

	**Day 1** ***n*** **=** **850**				**Day 7** ***n*** **=** **800**				**Day 14** ***n*** **=** **780**			
	**Observed proportion BPD/death** **(*n =* 502)**	**Mean predicted probability BPD/death**	**Calibration intercept** **(95% CI)**	**Calibration slope** **(95% CI)**	**Observed proportion BPD/death** **(*n =* 453)**	**Mean predicted probability BPD/death**	**Calibration intercept** **(95% CI)**	**Calibration slope** **(95% CI)**	**Observed proportion BPD/death** **(*n =* 433)**	**Mean predicted probability BPD/death**	**Calibration intercept (95% CI)**	**Calibration slope** **(95% CI)**
Standard Logistic Regression (LR)	0.591	0.588	0.01 (−0.14, 0.17)	1.08 (0.92, 1.25)	0.566	0.564	0.01 (−0.15, 0.17)	1.05 (0.89, 1.22)	0.555	0.546	0.05 (−0.11, 0.21)	1.00 (0.84, 1.16)
Penalized LR	0.591	0.588	0.01 (−0.14, 0.16)	1.11 (0.93, 1.28)	0.566	0.564	0.01 (−0.15, 0.17)	1.07 (0.90, 1.24)	0.555	0.547	0.05 (−0.12, 0.21)	1.05 (0.89, 1.22)
Support Vector Machine	0.591	0.594	−0.02 (−0.17, 0.13)	1.02 (0.86, 1.18)	0.566	0.563	0.01 (−0.14, 0.17)	0.99 (0.83, 1.15)	0.555	0.550	0.02 (−0.13, 0.18)	1.00 (0.85, 1.16)
K-Nearest Neighbor	0.591	0.582	0.05 (−0.11, 0.22)	0.07 (0.05, 0.09)	0.566	0.567	−0.003 (−0.18, 0.17)	0.07 (0.05, 0.09)	0.555	0.552	0.02 (−0.16, 0.19)	0.09 (0.07, 0.11)
Artificial Neural Network	0.591	0.678	−0.46 (−0.61, −0.31)	0.98 (0.86, 1.11)	0.566	0.621	−0.28 (−0.44, −0.13)	1.00 (0.84, 1.15)	0.555	0.569	−0.07 (−0.22, 0.09)	1.20 (1.00, 1.39)
Random Forest	0.591	0.584	0.04 (−0.13, 0.21)	0.39 (0.30, 0.47)	0.566	0.558	0.05 (−0.12, 0.22)	0.53 (0.42, 0.63)	0.555	0.544	0.06 (−0.11, 0.23)	0.69 (0.57, 0.81)
Soft Voting Ensemble	0.591	0.599	−0.04 (−0.19, 0.11)	1.06 (0.90, 1.23)	0.566	0.564	0.01 (−0.15, 0.17)	1.06 (0.89, 1.23)	0.555	0.554	0.003 (−0.16, 0.16)	1.09 (0.92, 1.26)
Stacking Neural Network Ensemble	0.591	0.569	0.10 (−0.05, 0.24)	1.49 (1.26, 1.73)	0.566	0.630	−0.29 (−0.44, −0.15)	1.40 (1.20, 1.60)	0.555	0.594	−0.19 (−0.34, −0.03)	1.21 (1.02, 1.39)

*NICU, Neonatal intensive care unit*.

We compared the summary performance statistics detailed above (AUC, calibration intercept and slope) to select the models that will be further explored when we develop an online tool for estimating BPD severity and death (as noted in the introduction). However, we have also provided data on additional performance measures (sensitivity, specificity, positive and negative predictive values, and loess-smoothed calibration curves) in Supplementary Tables 3, 4 and Supplementary Figure 1 for readers who are interested in a more detailed comparison of the models.

**Figure 2:**
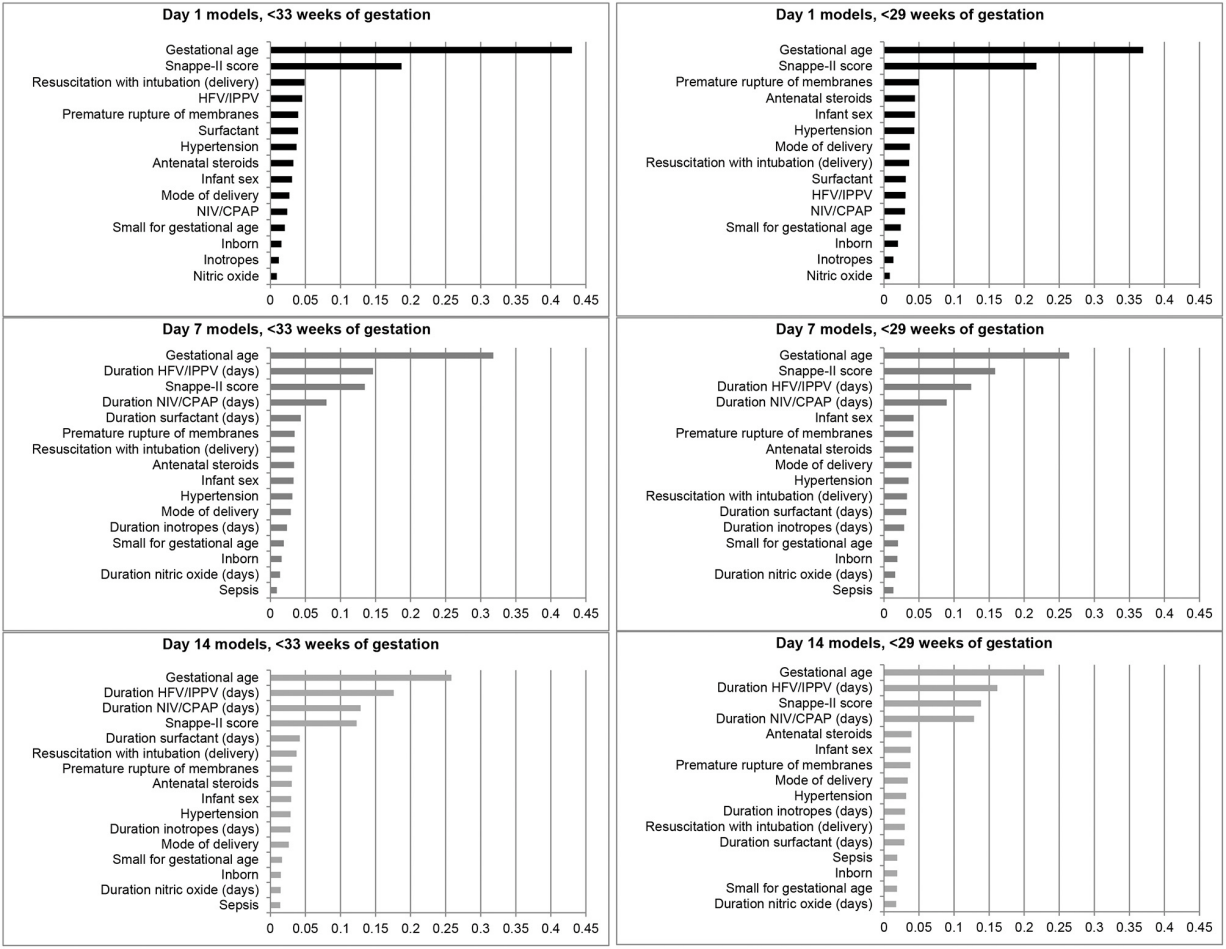
Feature importance plots illustrating relative importance of each variable in predicting the outcome of bronchopulmonary dysplasia or death prior to NICU discharge among infants born at <33 and <29 weeks of gestation in Canada, 2016–2018. HFV, High-frequency ventilation; IPPV, Intermittent positive pressure ventilation; NIV, Non-invasive ventilation; CPAP, Continuous positive airway pressure.

The feature importance plots (Figure 2) illustrate that GA was the most important predictor of BPD/death at all three time points in both cohorts. The SNAPPE-II score (Day 1, both cohorts; Day 7, <29 weeks' GA) and duration of high-frequency or intermittent positive pressure ventilation (Day 7, <33 weeks' GA; Day 14, both cohorts) were the second most influential predictors.

## Discussion

In our comparison of standard logistic regression and machine learning methods for predicting BPD/death, no one method/model clearly outperformed all the others. However, certain models performed less well: the AUCs for the random forest and k-nearest neighbor models were often the lowest, and their predictions were consistently too extreme (calibration slope <1). The AUCs for the artificial neural network and stacking neural network ensemble models were generally similar to those reported for the logistic regression models in the main cohort, but they were consistently lower in the <29 weeks' GA subcohort.

Moreover, the calibration performance of those models (artificial neural network, stacking neural network ensembles) was often suboptimal. A 2019 systematic review of 71 studies that compared machine learning and logistic regression models for predicting a variety of clinical outcomes highlighted the frequent failure on the part of investigators to report calibration performance (35). Models that exhibit good discrimination may still yield unreliable predicted risks (22), which could lead to systematic errors in decision-making (58). Assessing model calibration is particularly important with machine learning approaches: while logistic regression models can be recalibrated by reestimating the intercept (22) and updating the regression coefficients (51), methods for improving the calibration of machine learning models are “unclear” (59).

Machine learning is often touted for its superior ability to model complex, non-linear relationships (48, 60). It is possible that the relationship between the predictors we examined and the outcome of BPD/death is adequately captured by fitting standard logistic regression models, even without the inclusion of interaction terms. The ensemble models, which are designed to boost predictive accuracy (61), also failed to outperform logistic regression. While we did not empirically explore the reasons for this, the algorithms we tested may not have provided sufficient “diversity of opinion”— in other words, all the models may have tended to make similar predictions for the same individuals—for the ensemble methods to have been effective (61, 62). It is worth noting, however, that in Jaskari et al.'s comparison of nine machine learning and regression-based algorithms for predicting BPD, the random forest ensemble model had one of the best discrimination performances with an AUC of 0.884, compared to 0.856 for the logistic regression model (20).

As noted in the results section, we used the AUC and calibration intercept and slope to select the top-performing models, rather than comparing sensitivity, specificity or predictive values. Additionally, we did not, at this stage, consider how well the predicted probabilities aligned with the observed outcomes across the full predictive range. These decisions were made in consideration of the current aim, which was to select candidate algorithms by comparing summary performance measures for binary BPD prediction models. In the next phase of our work, we plan to use these algorithms to develop multinomial models for predicting BPD severity and death, and to translate those top-performing models into an online risk assessment tool. A comprehensive evaluation of model performance will be undertaken at that time that considers clinical relevance. For example, sensitivity may be the metric of choice for the Day 1 model, as the goal may be to provide minimal-risk therapies to as many at-risk infants as possible. In contrast, a high positive predictive value for the Day 14 model would help to ensure that more aggressive therapies are appropriately targeted at those who are truly at high risk.

While our study used population-based data from a cohort managed using contemporary respiratory support practices, our selection of variables was limited to those collected by the existing CNN database and we were unable to include some potentially important predictors in our models [e.g., FiO_2_ (13), diagnosis of patent ductus arteriosus on or before 7 days of age (63)]. Nevertheless, the logistic regression, support vector machine, and soft voting ensemble models demonstrated acceptable discrimination (≥0.83 at all prediction time points for infants born prior to 33 weeks of gestation; ≥0.74 for infants born prior to 29 weeks of gestation) and calibration (as determined by examining the calibration intercept and slope).

## Conclusion

Although none of the machine learning models in this study consistently outperformed standard logistic regression in predicting the binary outcome of BPD/death, we cannot assume this will be the case for the multinomial outcome of BPD severity (mild/moderate/severe) and death. Accordingly, we plan to apply a similar approach in the next phase of our work—developing multinomial models to predict BPD severity and death—by testing the most promising algorithms identified in this study, i.e., standard and penalized logistic regression, support vector machine, and soft voting ensemble.

While the comprehensive approach described in this paper may seem laborious, it avoids implicit assumptions about the optimal prediction algorithm and allows investigators to directly compare the predictive performance of multiple models fit using different algorithms. As such, this approach provides more confidence in the clinical prediction tools ultimately developed to inform clinical decision-making.

## Data Availability Statement

The data analyzed in this study are housed in a database maintained by the Canadian Neonatal Network. Current data sharing agreements do not allow these data to be shared publicly.

## Ethics Statement

The studies involving human participants were reviewed and approved by the Queen's University Health Sciences and Affiliated Teaching Hospitals Research Ethics Board and the Mount Sinai Hospital Research Ethics Board. Written informed consent for participation was not required for this study in accordance with the national legislation and the institutional requirements.

## Author Contributions

FK, HC, and PS contributed to the conception of the study. FK, HC, AK, JT, and PS were involved in the study design and the funding application. PS led the data acquisition. JM performed the data analysis with assistance from HC and AK. HC and JM drafted sections of the manuscript. All authors contributed to the interpretation of the data, critically reviewed the manuscript, and approved the submitted version.

## Funding

This work was supported by an operating grant from the Innovation Fund (ifpoc.org), a joint initiative of the Ontario Ministry of Health and the Ontario Medical Association. The funder had no role in the design or conduct of this study, or in the interpretation of the findings.

## Conflict of Interest

The authors declare that the research was conducted in the absence of any commercial or financial relationships that could be construed as a potential conflict of interest.

## Publisher's Note

All claims expressed in this article are solely those of the authors and do not necessarily represent those of their affiliated organizations, or those of the publisher, the editors and the reviewers. Any product that may be evaluated in this article, or claim that may be made by its manufacturer, is not guaranteed or endorsed by the publisher.
