# Supplementary material for: Comparison of Multivariable Logistic Regression and Machine Learning Models for Predicting Bronchopulmonary Dysplasia or Death in Very Preterm Infants
Source: Front Pediatr. 2021 Dec 7;9:759776. doi: 10.3389/fped.2021.759776 (PMC8688959; doi:10.3389/fped.2021.759776)
Supplement: Supplementary file 1 [file Data_Sheet_1.docx]

**Supplementary Table 1.** Description of machine learning algorithms used to develop and compare models for predicting bronchopulmonary dysplasia/death prior to NICU discharge and hyperparameter settings

| **Algorithm/ensemble** | **Hyperparameter** | **Description** | **Value** |
| --- | --- | --- | --- |
| Penalized logistic regression | Penalty | Differentiates “standard” logistic regression algorithm from penalized logistic regression | L2 ridge penalty |
|  | Class_weight | Applies weights to outcome classes | None |
|  | Solver | Specifies algorithm used to modify model weights during training | ‘lbfgs’ |
|  | Max_iter | Limits number of iterations before solver converges | 400 |
|  | Multi_class | Specifies whether target is binary or multiclass | ‘ovr’ |
| Support vector machine | C | Regularization parameter | 1.0 |
|  | kernel | Kernel type | ‘rbf’ (radial basis function) |
|  | probability | Enables output of probability estimates | True |
| k-nearest neighbor | N_neighbors | Specifies number of neighboring points used to make prediction | 5 |
|  | Weights | Specifies whether different neighbors more strongly influence the prediction. We gave all n_neighbors equal weight. | Uniform |
|  | P | Algorithm which calculates distance between points. Power parameter for the Minkowski metric: 1=Manhattan distance, 2= Euclidean | 2 |
| Artificial neural network | Input layer | First layer of the neural network; specifies number of neurons and shape of dataset | Input was the number of features and the number of neurons was set to 200. Activation function was ‘relu’. |
|  | Hidden layers | Number of layers between the input and output layers | 4 hidden layers with an hourglass shape. The first and last layer had 50 neurons and the middle two layers had 10 neurons. Activation function was ‘relu’. |
|  | Output layer | Final layer outputting the prediction | Prediction was a single value, so the output only has 1 neuron. Activation function was ‘sigmoid’. |
|  | Optimizer | Algorithm used to modify model weights during training | ‘adam’ |
|  | Loss function | Used to determine how well the above weights are performing | ‘binary_crossentropy’ |
| Random forest | N_estimators | Random forest is an ensemble of decision trees; n_estimators specifies the number of trees in the forest. | 500 |
|  | Max_depth | Specifies maximum number of layers in trees | None |
|  | Max_features | Limits the number of predictors used | ‘auto’ |
|  | Max_leaf_notes | Limits the number of leaf nodes when splitting | None |
|  | Bootstrap | Specifies whether bootstrap samples should be used to build the trees rather than the entire dataset | True |
|  | Max_samples | If “Bootstrap” is specified, limits the number of samples for training each tree | None |
|  | Min_sample_leafs | Number of samples required for leaf nodes | 2 |
| Soft voting ensemble | Estimators | With soft voting ensembles, each constituent model’s predicted probabilities of class membership are summed and compared. In our case, the predicted probabilities of “BPD/death” and “Survived to NICU discharge without BPD” output by six models (penalized and standard logistic regression, support vector machine, k-nearest neighbor, artificial neural network, and random forest) were summed. The outcome class with the highest sum formed the soft voting ensemble’s predicted outcome. | All models |
|  | Voting | Type of voting: **Hard** voting uses each model’s predicted outcome.  **Soft** voting uses each model’s predicted probabilities. | Soft |
| Stacking neural network ensemble | Layers, optimizer, and loss function | Stacking refers to an ensemble method whereby a “meta model” is trained using the predictions from multiple heterogenous models. In our case, the predicted probabilities output by the same six models used in the soft voting ensemble formed the predictors when training the stacking neural network ensemble models. (In other words, rather than the predictors shown in Table 1, the predicted probabilities of BPD/death were used as the predictor variables.) | Number of inputs was equal to the number of models in the ensemble instead of the shape of the dataset. Everything else was identical to the artificial neural network model. |

**Supplementary Table 2.** Area under the curve^a^ (AUC) for models predicting bronchopulmonary dysplasia or death prior to NICU discharge at three time points (Days 1, 7 and 14 of NICU stay) among infants born at <33 weeks and <29 weeks of gestation who were admitted to Canadian tertiary-care NICUs, 2016–2018, with gestational age modelled as continuous variable

| **Model** | **<33 weeks** | | | **<29 weeks** | | |
| --- | --- | --- | --- | --- | --- | --- |
|  | **Day 1** | **Day 7** | **Day 14** | **Day 1** | **Day 7** | **Day 14** |
| Standard Logistic Regression (LR) | 0.860 | 0.885 | 0.878 | 0.778 | 0.781 | 0.781 |
| Penalized LR | 0.860 | 0.884 | 0.882 | 0.776 | 0.783 | 0.782 |
| Support Vector Machine | 0.835 | 0.859 | 0.852 | 0.752 | 0.777 | 0.767 |
| K-Nearest Neighbor | 0.797 | 0.836 | 0.830 | 0.692 | 0.737 | 0.736 |
| Artificial Neural Network | 0.862 | 0.882 | 0.878 | 0.754 | 0.783 | 0.770 |
| Random Forest | 0.814 | 0.858 | 0.859 | 0.715 | 0.751 | 0.749 |
| Soft Voting Ensemble | 0.856 | 0.885 | 0.883 | 0.769 | 0.781 | 0.781 |
| Stacking Neural Network Ensemble | 0.858 | 0.887 | 0.882 | 0.768 | 0.775 | 0.775 |

NICU: Neonatal intensive care unit

^a^ Average from 10-fold cross-validation procedure

**Supplementary Table 3.** Sensitivity, specificity, positive predictive value (PPV) and negative predictive value (NPV) for models predicting bronchopulmonary dysplasia or death prior to NICU discharge at three time points (Days 1, 7 and 14 of NICU stay) among infants born at **<33** weeks of gestation who were admitted to Canadian tertiary-care NICUs, 2016–2018

| **Model** | **Day 1** | | | | **Day 7** | | | | **Day 14** | | | |
| --- | --- | --- | --- | --- | --- | --- | --- | --- | --- | --- | --- | --- |
|  | **Sensitivity, %**  **(95% CI)** | **Specificity, %**  **(95% CI)** | **PPV, %**  **(95% CI)** | **NPV, %**  **(95% CI)** | **Sensitivity, %**  **(95% CI)** | **Specificity, %**  **(95% CI)** | **PPV, %**  **(95% CI)** | **NPV, %**  **(95% CI)** | **Sensitivity, %**  **(95% CI)** | **Specificity, %**  **(95% CI)** | **PPV, %**  **(95% CI)** | **NPV, %**  **(95% CI)** |
| Standard Logistic Regression (LR) | 60.66  (56.75-64.47) | 90.29  (88.45-91.93) | 77.40  (73.98-80.49) | 80.72  (79.15-82.20) | 64.08  (60.02-67.99) | 90.72  (88.91-92.33) | 77.45  (73.97-80.59) | 83.54  (81.97-85.01) | 59.78  (55.58-63.89) | 89.86  (87.98-91.54) | 73.84  (70.13-77.23) | 82.36  (80.81-83.81) |
| Penalized LR | 60.66  (56.75-64.47) | 90.03  (88.17-91.70) | 76.94  (73.52-80.03) | 80.68  (79.10-82.16) | 64.08  (60.02-67.99) | 90.81  (89.00-92.41) | 77.62  (74.13-80.75) | 83.56  (81.99-85.02) | 59.96  (55.76-64.06) | 89.78  (87.89-91.46) | 73.73  (70.03-77.12) | 82.41  (80.86-83.86) |
| Support Vector Machine | 61.91  (58.02-65.70) | 90.89  (89.09-92.48) | 78.84  (75.47-81.86) | 81.32  (79.75-82.80) | 59.93  (55.81-63.95) | 91.84  (90.11-93.35) | 78.51  (74.87-81.75) | 82.17  (80.64-83.60) | 56.91  (52.68-61.07) | 91.58  (89.84-93.11) | 76.39  (72.53-79.85) | 81.62  (80.12-83.03) |
| K-Nearest Neighbor | 60.03  (56.11-63.86) | 86.43  (84.32-88.34) | 70.79  (67.42-73.95) | 79.78  (78.15-81.31) | 59.76  (55.64-63.78) | 86.77  (84.69-88.66) | 69.20  (65.65-72.53) | 81.26  (79.66-82.76) | 54.76  (50.52-58.95) | 87.97  (85.96-89.79) | 68.54  (64.70-72.14) | 80.25  (78.72-81.70) |
| Artificial Neural Network | 53.92  (49.96-57.84) | 92.18  (90.49-93.66) | 79.08  (75.40-82.34) | 78.49  (77.01-79.90) | 60.97  (56.86-64.96) | 92.35  (90.67-93.81) | 79.86  (76.27-83.03) | 82.63  (81.10-84.06) | 53.32  (49.08-57.53) | 92.96  (91.33-94.36) | 78.36  (74.35-81.90) | 80.63  (79.18-82.00) |
| Random Forest | 62.23  (58.34-66.00) | 85.48  (83.32-87.46) | 70.14  (66.87-73.22) | 80.50  (78.84-82.06) | 65.11  (61.07-69.00) | 87.37  (85.33-89.23) | 71.95  (68.55-75.11) | 83.43  (81.80-84.94) | 65.53  (61.42-69.47) | 87.37  (85.33-89.23) | 71.29  (67.85-74.50) | 84.12  (82.50-85.62) |
| Soft Voting Ensemble | 63.17  (59.29-66.92) | 89.52  (87.62-91.22) | 76.76  (73.44-79.79) | 81.60  (79.99-83.10) | 64.59  (60.55-68.49) | 90.72  (88.91-92.33) | 77.59  (74.13-80.72) | 83.74  (82.17-85.21) | 59.07  (54.85-63.18) | 91.92  (90.21-93.43) | 77.78  (74.02-81.13) | 82.43  (80.92-83.85) |
| Stacking Neural Network Ensemble | 63.95  (60.09-67.68) | 89.26  (87.34-90.98) | 76.55  (73.25-79.55) | 81.88  (80.26-83.39) | 54.06  (49.90-58.17) | 94.33  (92.84-95.59) | 82.59  (78.76-85.85) | 80.50  (79.06-81.86) | 67.15  (63.07-71.04) | 87.46  (85.42-89.31) | 71.92  (68.53-75.08) | 84.76  (83.14-86.26) |

NICU: Neonatal intensive care unit

**Supplementary Table 4.** Sensitivity, specificity, positive predictive value (PPV) and negative predictive value (NPV) for models predicting bronchopulmonary dysplasia or death prior to NICU discharge at three time points (Days 1, 7 and 14 of NICU stay) among infants born at **<29** weeks of gestation who were admitted to Canadian tertiary-care NICUs, 2016–2018

| **Model** | **Day 1** | | | | **Day 7** | | | | **Day 14** | | | |
| --- | --- | --- | --- | --- | --- | --- | --- | --- | --- | --- | --- | --- |
|  | **Sensitivity, %**  **(95% CI)** | **Specificity, %**  **(95% CI)** | **PPV, %**  **(95% CI)** | **NPV, %**  **(95% CI)** | **Sensitivity, %**  **(95% CI)** | **Specificity, %**  **(95% CI)** | **PPV, %**  **(95% CI)** | **NPV, %**  **(95% CI)** | **Sensitivity, %**  **(95% CI)** | **Specificity, %**  **(95% CI)** | **PPV, %**  **(95% CI)** | **NPV, %**  **(95% CI)** |
| Standard Logistic Regression (LR) | 76.29  (72.33-79.95) | 64.08  (58.79-69.13) | 75.39  (72.53-78.04) | 65.20  (61.12-69.07) | 73.51  (69.19-77.52) | 68.30  (63.12-73.16) | 75.17  (71.98-78.10) | 66.39  (62.51-70.05) | 72.06  (67.57-76.23) | 70.32  (65.21-75.08) | 75.18  (71.83-78.25) | 66.85  (63.07-70.42) |
| Penalized LR | 76.29  (72.33-79.95) | 63.22  (57.91-68.30) | 74.95  (72.11-77.59) | 64.90  (60.79-68.80) | 73.51  (69.19-77.52) | 67.44  (62.23-72.34) | 74.66  (71.50-77.59) | 66.10  (62.20-69.80) | 71.59  (67.09-75.80) | 71.18  (66.11-75.89) | 75.61  (72.23-78.70) | 66.76  (63.03-70.29) |
| Support Vector Machine | 77.89  (74.00-81.45) | 59.20  (53.83-64.41) | 73.36  (70.64-75.91) | 64.98  (60.65-69.09) | 71.08  (66.67-75.22) | 68.59  (63.42-73.44) | 74.71  (71.44-77.72) | 64.50  (60.73-68.09) | 72.98  (68.53-77.11) | 68.30  (63.12-73.16) | 74.18  (70.90-77.21) | 66.95  (63.07-70.61) |
| K-Nearest Neighbor | 75.10  (71.08-78.82) | 58.05  (52.67-63.29) | 72.08  (69.32-74.69) | 61.77  (57.54-65.84) | 71.74  (67.35-75.85) | 58.50  (53.12-63.74) | 69.30  (66.29-72.14) | 61.33  (57.19-65.31) | 69.75  (65.18-74.04) | 60.23  (54.87-65.42) | 68.64  (65.47-71.64) | 61.47  (57.46-65.33) |
| Artificial Neural Network | 74.50  (70.45-78.26) | 62.36  (57.03-67.47) | 74.06  (71.19-76.74) | 62.90  (58.84-66.78) | 72.63  (68.27-76.68) | 66.86  (61.63-71.79) | 74.10  (70.92-77.05) | 65.17  (61.28-68.86) | 78.29  (74.11-82.08) | 59.37  (53.99-64.58) | 70.62  (67.72-73.38) | 68.67  (64.24-72.78) |
| Random Forest | 71.71  (67.55-75.61) | 60.06  (54.70-65.24) | 72.14  (69.24-74.87) | 59.54  (55.55-63.41) | 72.19  (67.81-76.27) | 61.10  (55.74-66.25) | 70.78  (67.72-73.66) | 62.72  (58.66-66.61) | 75.29  (70.95-79.28) | 64.84  (59.56-69.86) | 72.77  (69.64-75.69) | 67.77  (63.68-71.61) |
| Soft Voting Ensemble | 76.29  (72.33-79.95) | 60.34  (54.99-65.52) | 73.51  (70.73-76.12) | 63.83  (59.61-67.84) | 73.73  (69.42-77.73) | 67.72  (62.52-72.62) | 74.89  (71.72-77.81) | 66.38  (62.48-70.08) | 70.67  (66.13-74.92) | 72.33  (67.31-76.98) | 76.12  (72.68-79.25) | 66.40  (62.74-69.87) |
| Stacking Neural Network Ensemble | 87.65  (84.45-90.40) | 44.54  (39.24-49.93) | 69.51  (67.36-71.58) | 71.43  (65.82-76.44) | 76.38  (72.19-80.22) | 62.25  (56.92-67.37) | 72.54  (69.57-75.32) | 66.87  (62.66-70.83) | 55.66  (50.84-60.40) | 85.30  (81.13-88.86) | 82.53  (78.35-86.06) | 60.66  (57.90-63.35) |

NICU: Neonatal intensive care unit

Supplementary Figure 1. Loess-based calibration curves for models predicting BPD or death prior to NICU discharge at three time points (Days 1, 7 and 14 of NICU stay) among infants admitted to Canadian tertiary-care NICUs, 2016–2018

| Day 1 models, cohort born at <33 weeks of gestation | 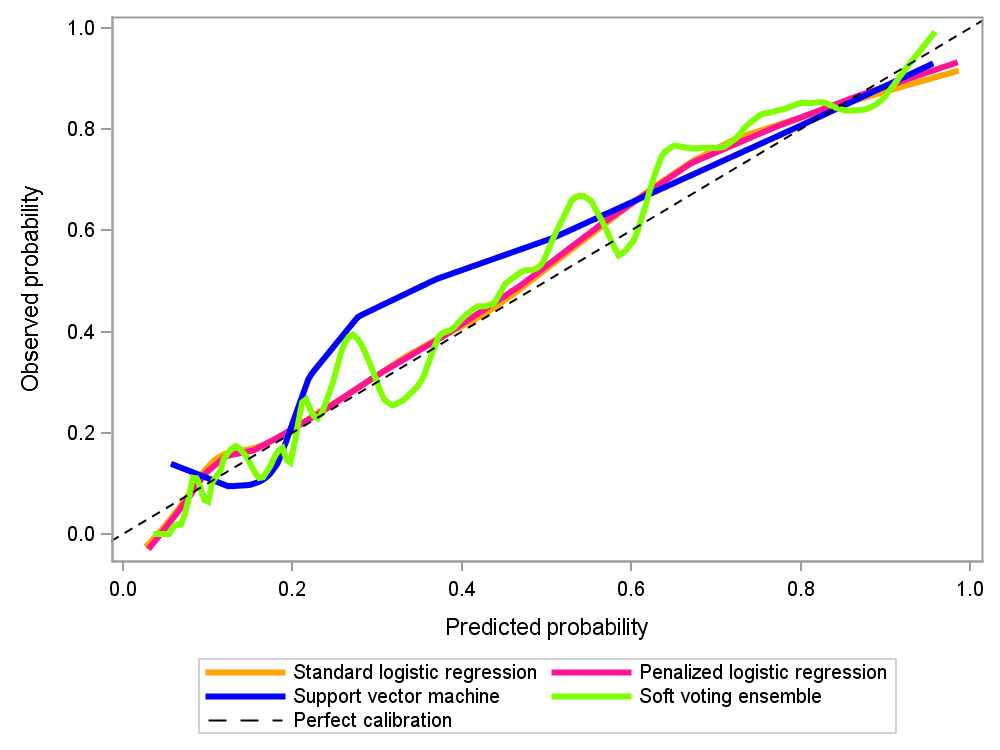 | 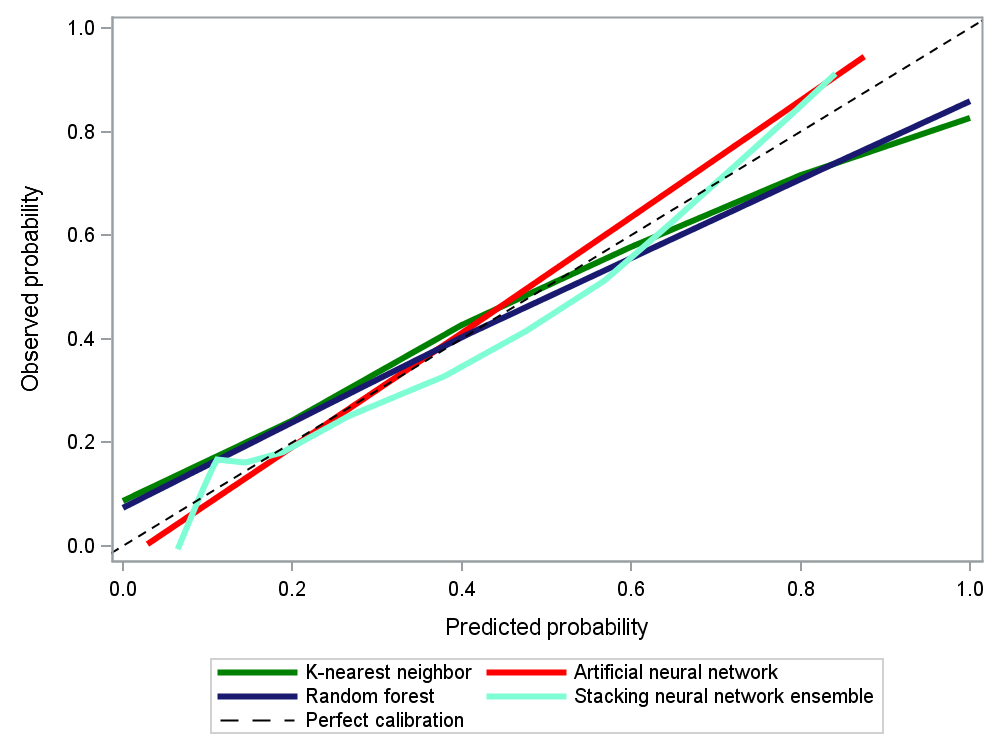 |
| --- | --- | --- |
| Day 7 models, cohort born at <33 weeks of gestation | 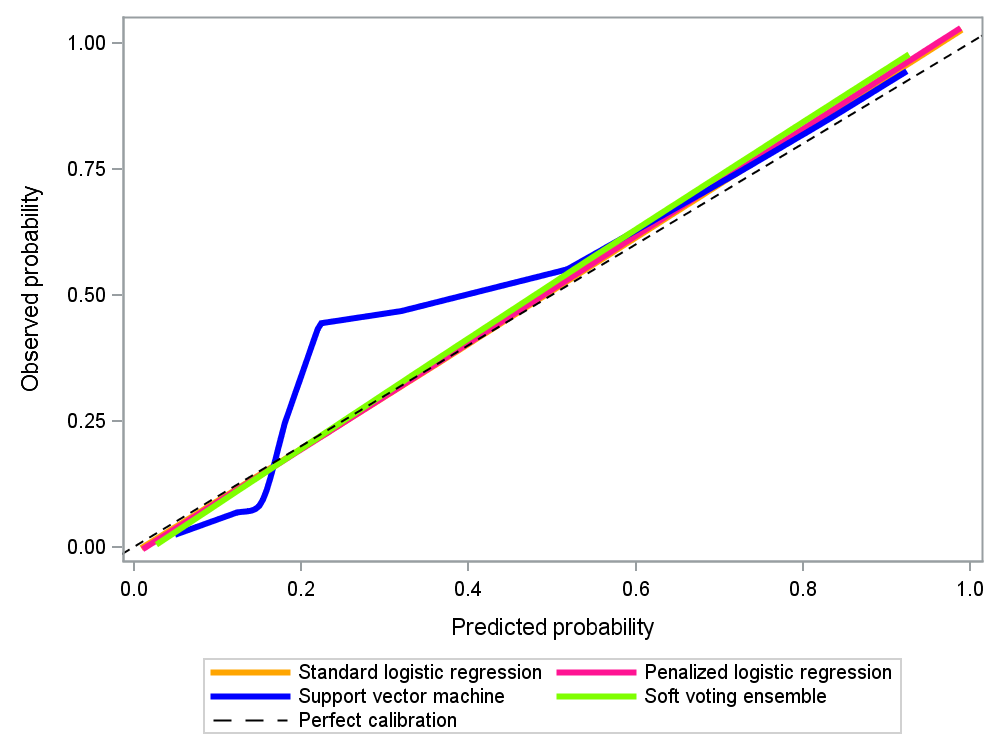 | 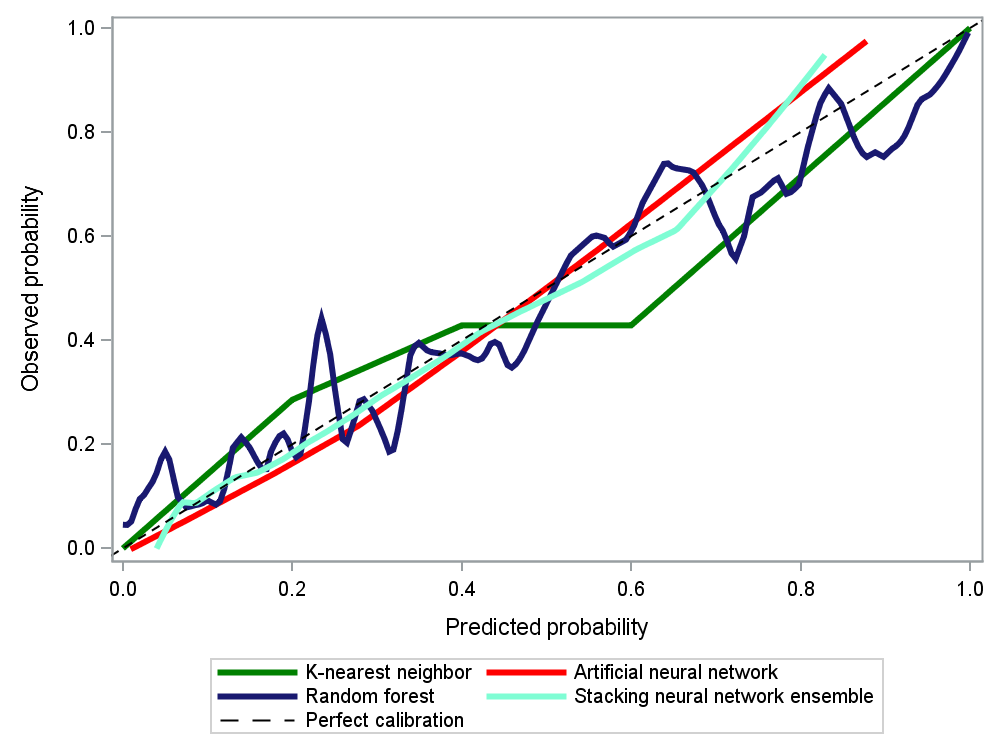 |
| Day 14 models, cohort born at <33 weeks of gestation | 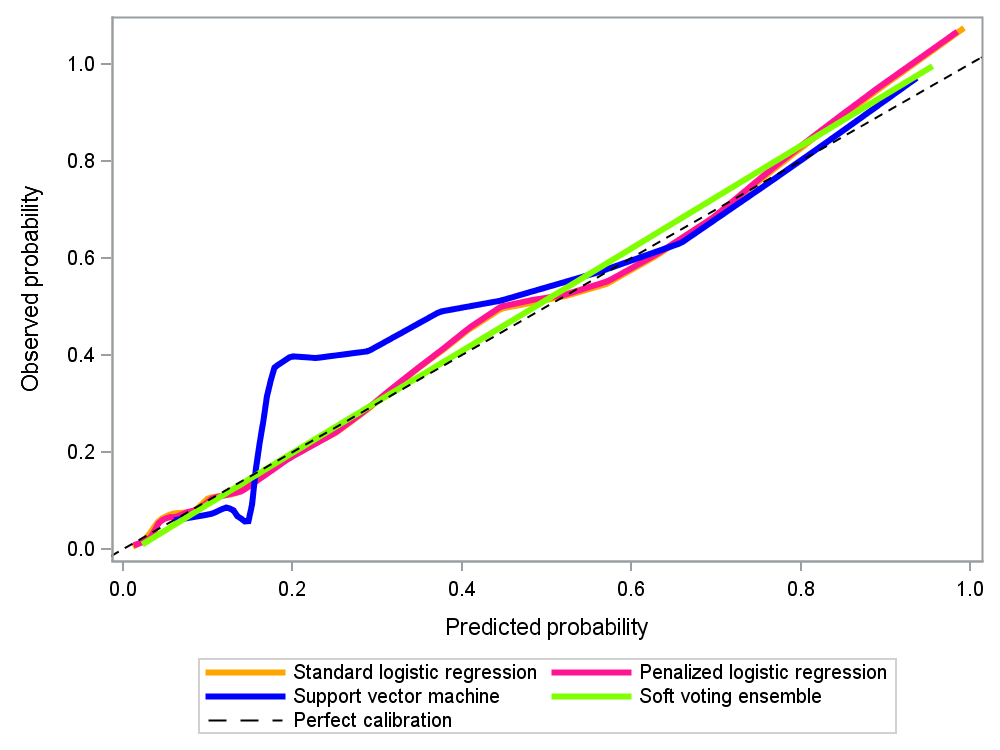 | 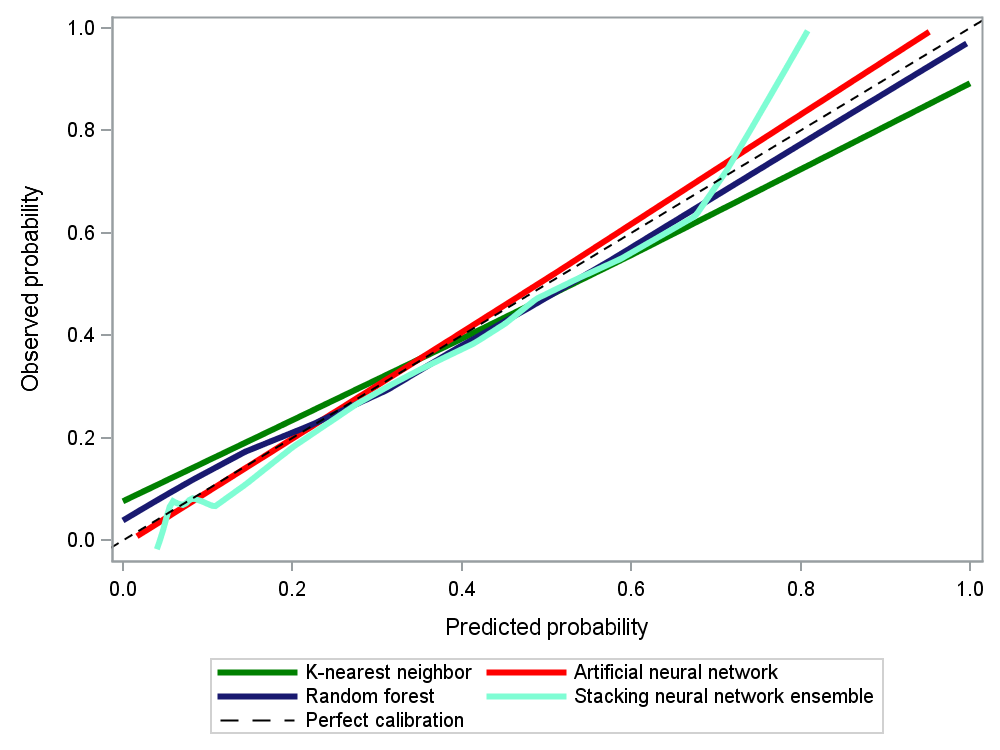 |
| Day 1 models, cohort born at <29 weeks of gestation | 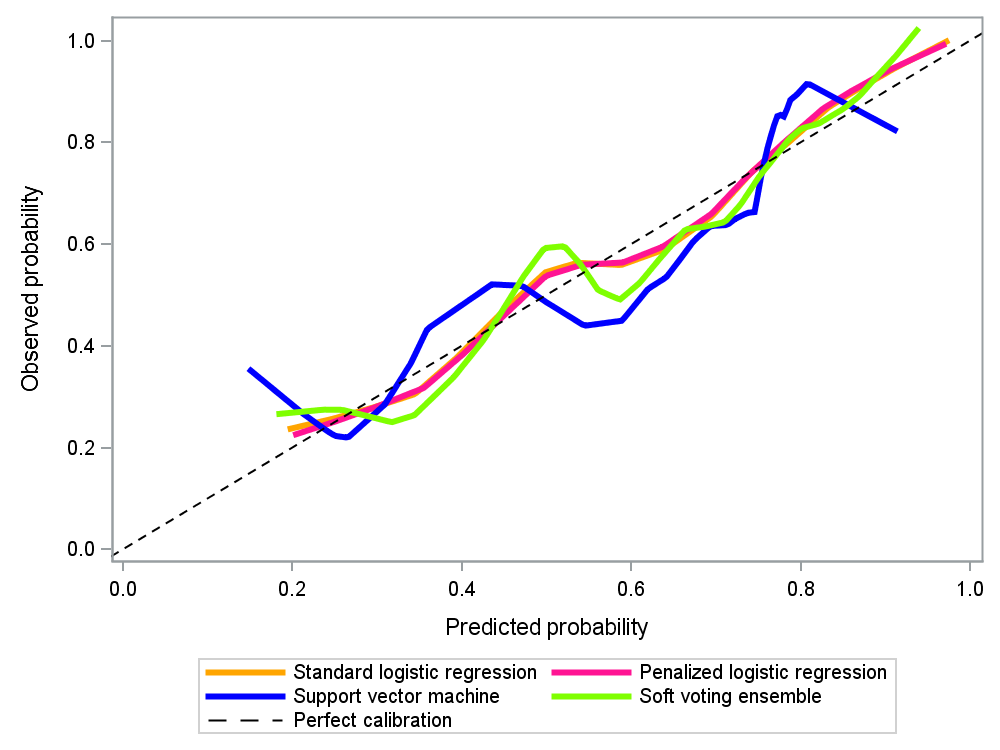 | 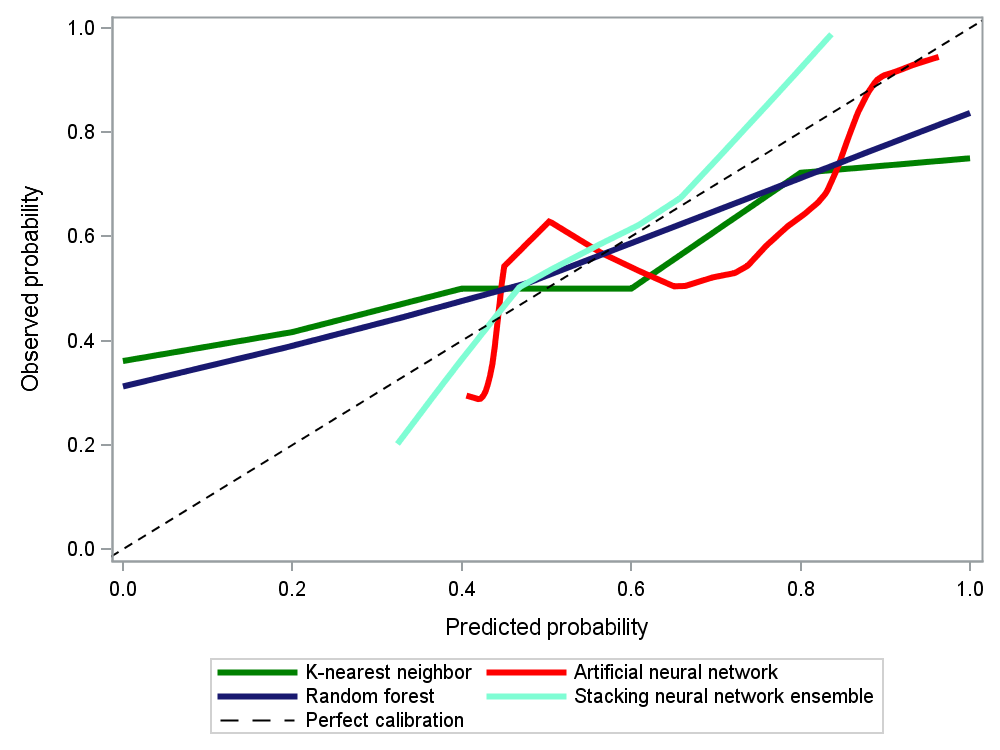 |
| Day 7 models, cohort born at <29 weeks of gestation | 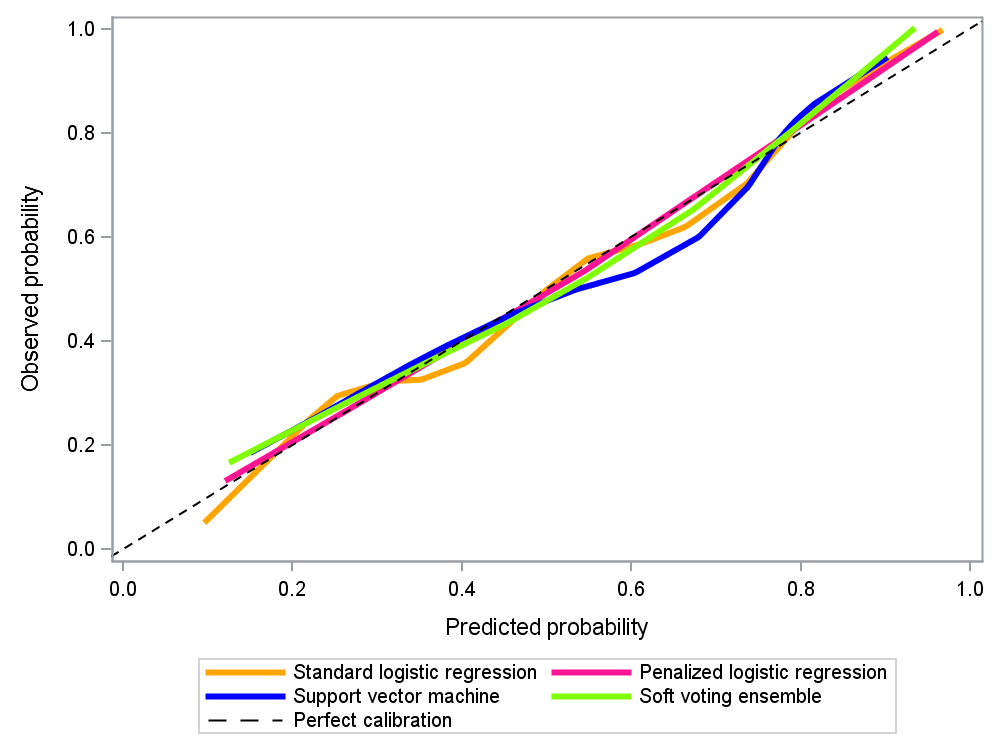 | 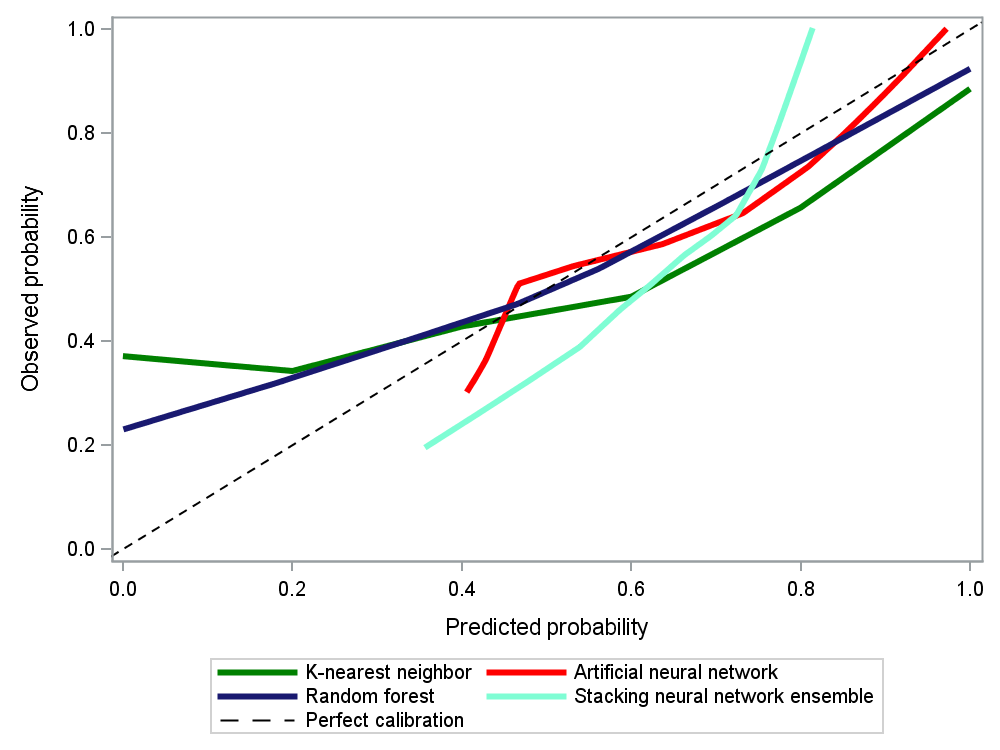 |
| Day 14 models, cohort born at <29 weeks of gestation | 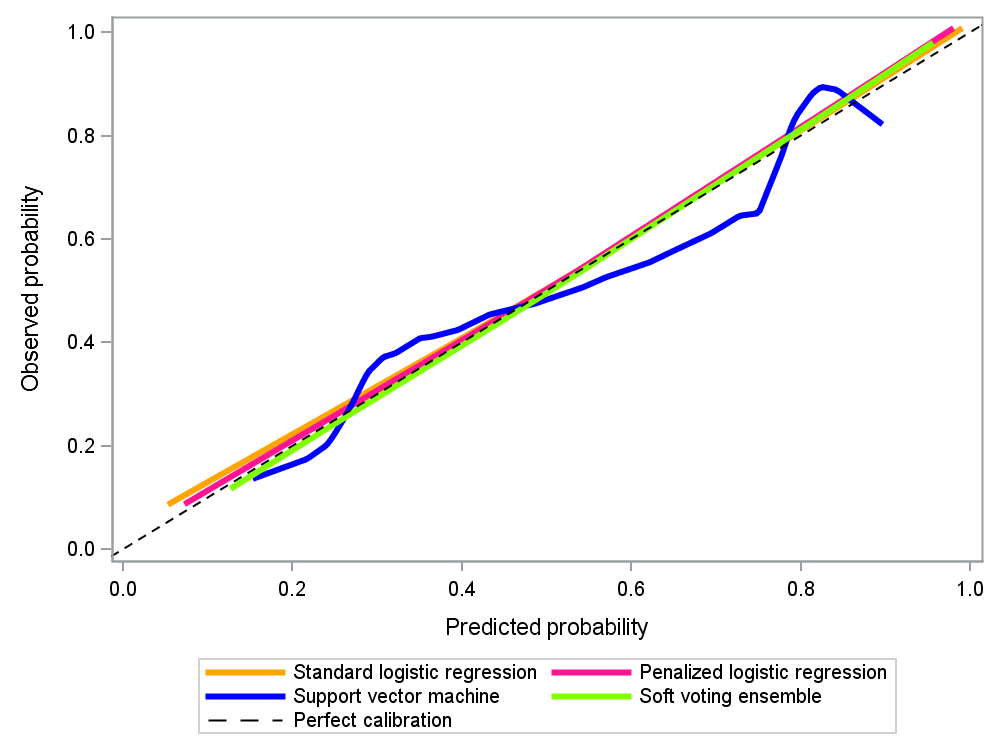 | 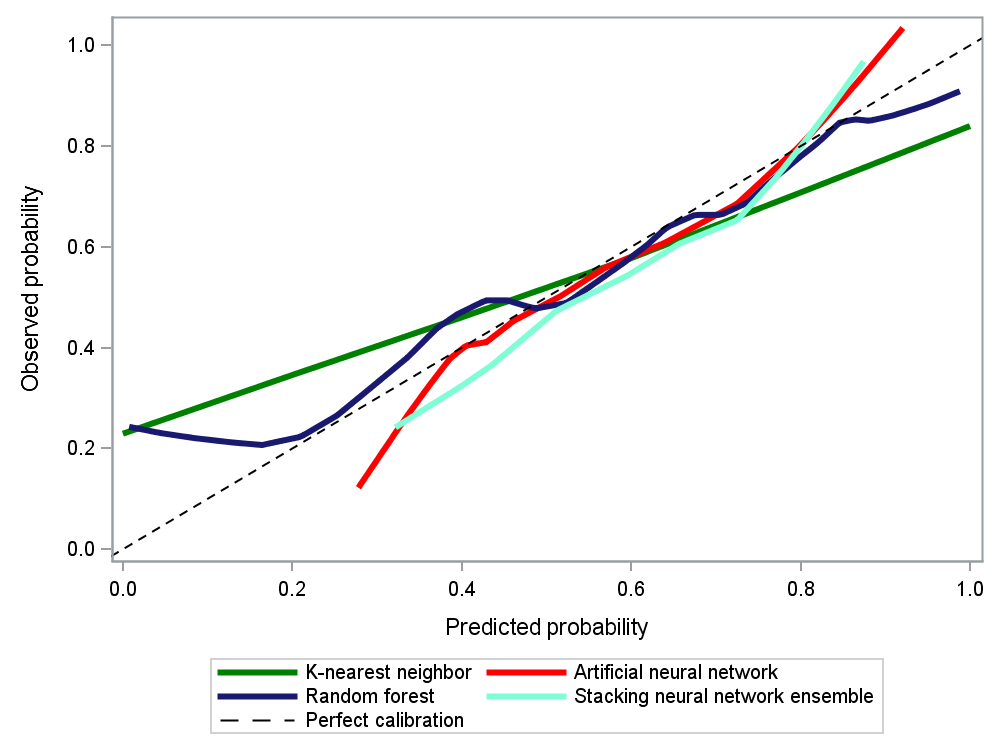 |

**CNN Site Investigators:** Prakesh S Shah, MD, MSc (Director, Canadian Neonatal Network and Site Investigator), Mount Sinai Hospital, Toronto, Ontario; Marc Beltempo, MD, (Associate Director, Canadian Neonatal Network and Site Investigator), Montreal Children’s Hospital at McGill University Health Centre, Montréal, Québec; Jaideep Kanungo, MD, Victoria General Hospital, Victoria, British Columbia; Joseph Ting, MD, British Columbia Women’s Hospital, Vancouver, British Columbia; Zenon Cieslak, MD, Royal Columbian Hospital, New Westminster, British Columbia; Rebecca Sherlock, MD, Surrey Memorial Hospital, Surrey, British Columbia; Ayman Abou Mehrem, MD, Foothills Medical Centre, Calgary, Alberta; Jennifer Toye, MD, and Khalid Aziz, MBBS, Royal Alexandra Hospital, Edmonton, Alberta; Carlos Fajardo, MD, Alberta Children’s Hospital, Calgary, Alberta; Jaya Bodani, MD, Regina General Hospital, Regina, Saskatchewan; Lannae Strueby, MD, Royal University Hospital, Saskatoon, Saskatchewan; Mary Seshia, MBChB, and Deepak Louis, MD, Winnipeg Health Sciences Centre, Winnipeg, Manitoba; Ruben Alvaro, MD, St. Boniface General Hospital, Winnipeg, Manitoba; Amit Mukerji, MD, Hamilton Health Sciences Centre, Hamilton, Ontario; Orlando Da Silva, MD, MSc, London Health Sciences Centre, London, Ontario; Sajit Augustine, MD, Windsor Regional Hospital, Windsor, Ontario; Kyong-Soon Lee, MD, MSc, Hospital for Sick Children, Toronto, Ontario; Eugene Ng, MD, Sunnybrook Health Sciences Centre, Toronto, Ontario; Brigitte Lemyre, MD, The Ottawa Hospital, Ottawa, Ontario; Thierry Daboval, MD, Children’s Hospital of Eastern Ontario, Ottawa, Ontario; Faiza Khurshid, MD, Kingston General Hospital, Kingston, Ontario; Victoria Bizgu, MD, Jewish General Hospital, Montréal, Québec; Keith Barrington, MBChB, Anie Lapoint, MD, and Guillaume Ethier, NNP, Hôpital Sainte-Justine, Montréal, Québec; Christine Drolet, MD, and Bruno Piedboeuf, MD, Centre Hospitalier Universitaire de Québec, Sainte Foy, Québec; Martine Claveau, MSc, LLM, NNP, Montreal Children’s Hospital at McGill University Health Centre, Montréal, Québec; Marie St-Hilaire, MD, Hôpital Maisonneuve-Rosemont, Montréal, Québec; Valerie Bertelle, MD, and Edith Masse, MD, Centre Hospitalier Universitaire de Sherbrooke, Sherbrooke, Québec; Roderick Canning, MD, Moncton Hospital, Moncton, New Brunswick; Hala Makary, MD, Dr. Everett Chalmers Hospital, Fredericton, New Brunswick; Cecil Ojah, MBBS, and Luis Monterrosa, MD, Saint John Regional Hospital, Saint John, New Brunswick; Julie Emberley, MD, Janeway Children’s Health and Rehabilitation Centre, St. John’s, Newfoundland; Jehier Afifi, MB BCh, MSc, IWK Health Centre, Halifax, Nova Scotia; Andrzej Kajetanowicz, MD, Cape Breton Regional Hospital, Sydney, Nova Scotia; Shoo K Lee, MBBS, PhD (Chairman, Canadian Neonatal Network), Mount Sinai Hospital, Toronto, Ontario.
